# Dysregulated APOBEC3G causes DNA damage and promotes genomic instability in multiple myeloma

**DOI:** 10.1038/s41408-021-00554-9

**Published:** 2021-10-08

**Authors:** Srikanth Talluri, Mehmet K. Samur, Leutz Buon, Subodh Kumar, Lakshmi B. Potluri, Jialan Shi, Rao H. Prabhala, Masood A. Shammas, Nikhil C. Munshi

**Affiliations:** 1 Dana Farber Cancer Institute, Boston, MA 02115 USA; 2 Veterans Administration Boston Healthcare System, West Roxbury, MA 02132 USA; 3 Harvard Medical School, Boston, MA 02215 USA

**Keywords:** Myeloma, Cancer genomics

## Abstract

Multiple myeloma (MM) is a heterogeneous disease characterized by significant genomic instability. Recently, a causal role for the AID/APOBEC deaminases in inducing somatic mutations in myeloma has been reported. We have identified APOBEC/AID as a prominent mutational signature at diagnosis with further increase at relapse in MM. In this study, we identified upregulation of several members of APOBEC3 family (A3A, A3B, A3C, and A3G) with A3G, as one of the most expressed APOBECs. We investigated the role of APOBEC3G in MM and observed that A3G expression and APOBEC deaminase activity is elevated in myeloma cell lines and patient samples. Loss-of and gain-of function studies demonstrated that APOBEC3G significantly contributes to increase in DNA damage (abasic sites and DNA breaks) in MM cells. Evaluation of the impact on genome stability, using SNP arrays and whole genome sequencing, indicated that elevated APOBEC3G contributes to ongoing acquisition of both the copy number and mutational changes in MM cells over time. Elevated APOBEC3G also contributed to increased homologous recombination activity, a mechanism that can utilize increased DNA breaks to mediate genomic rearrangements in cancer cells. These data identify APOBEC3G as a novel gene impacting genomic evolution and underlying mechanisms in MM.

## Introduction

Multiple myeloma (MM) is a plasma cell malignancy associated with a marked genomic instability. Whole exome and whole-genome sequencing analyses indicate a complex mutational spectrum [1–7]. The clonal heterogeneity plays a critical role in development of resistance to treatment and relapse [8, 9]. The high DNA damage and dysregulated repair are among important factors contributing to genomic instability. Previous data has established the role of dysregulated homologous recombination (HR) activity in the ongoing genomic rearrangements and instability in MM [1]. Recently, a role for the AID/APOBEC family of cytidine deaminases in generation of somatic mutations in cancer has been proposed, and APOBEC signature mutations have been identified in a variety of human cancers [2]. Data from our and other laboratories have also demonstrated that APOBEC mutational signature is prevalent in MM cell genome [3–6]. Furthermore, the APOBEC mutational signature correlates with sub-clonal diversity in myeloma [4, 7].

The APOBEC family of proteins is comprised of AID (activity induced deaminase) and 10 related APOBEC enzymes (A1, A2, A3A, A3B, A3C, A3D, A3F, A3G, A3H, and A4) [8, 9]. AID has been well studied for its role in somatic hyper mutation and class switch recombination of immunoglobulin genes. On the other hand, APOBECs (apolipoprotein B mRNA editing enzyme and catalytic polypeptide-like) have been shown to have roles in mRNA editing and in anti-viral immunity. However, a detailed understanding of the specific biological roles of each of these proteins is still unclear. Functionally, they can deaminate cytosine/deoxycytosine to uracil/deoxyuridine. Deamination of cytidine residues by APOBECs primarily occurs in single-stranded DNA (ssDNA) that is exposed during replication, transcription, or during DNA damage repair. Dysregulated activity of APOBECs has been shown to cause C>T transitions or C>G, C>A transversions in the DNA. In somatic cells, such mutations can facilitate oncogenesis.

Recently, a role for APOBECs in inducing mutations in cancer has been proposed [9]. A number of cancers including breast, lung, bladder, cervix and head and neck have been shown to frequently display mutational signatures attributed to the deoxycytidine deamination activity of one or more APOBEC enzymes [9, 10]. A3B has been shown to be the source of mutations in breast cancer and is upregulated in several different cancers and can predict treatment respose [11–18]. Interestingly, APOBECs seems to be more active later in tumor evolution, as seen by enrichment of these mutational signatures in sub-clonal cancer gene mutations in breast, head/neck squamous, bladder, and lung carcinoma [9]. The preferred target regions for APOBEC-induced mutations seem to be ssDNA intermediates that are produced during DNA replication, and repair and transcription [9, 19]. We and others have identified the prevalence of APOBEC signature associated mutations in myeloma which increases with disease progression [2, 20]. APOBEC mutational signatures are found to be associated with high mutational load and MAF/MAFB translocations that correlate with poor prognosis in myeloma [6].

In this study, we demonstrate that multiple APOBECs are highly expressed in MM. The impact of APOBEC3G (A3G), one of the most expressed APOBECs in MM, is further investigated in detail to report its influence on various parameters of genome stability in MM. These data identify A3G as a novel gene involved in increased DNA damage and dysregulation of DNA repair and genome stability.

## Results

### ABOBEC3G is dysregulated in multiple myeloma

**Fig. 1:**
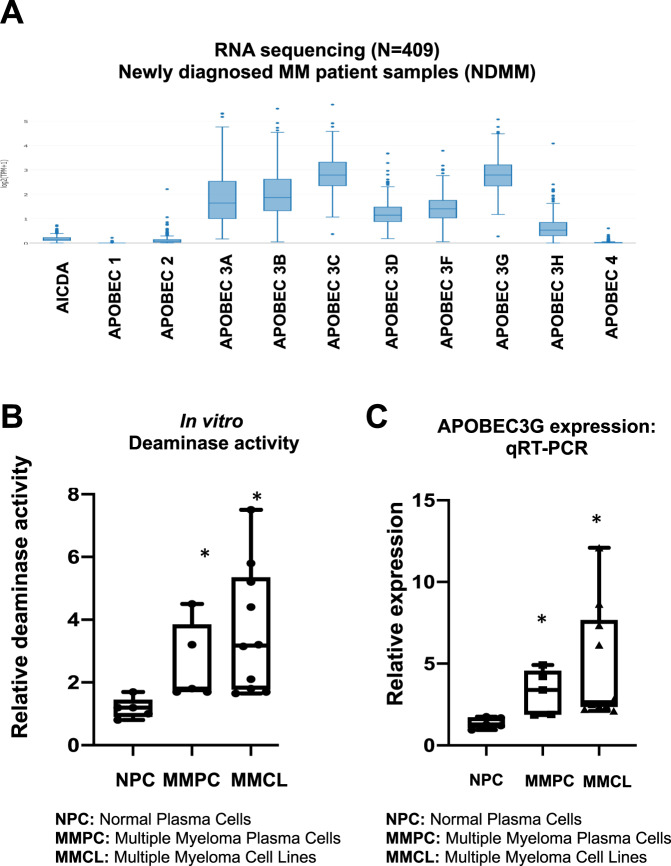
APOBEC expression and activity is dysregulated in Multiple Myeloma. **A** Expression of AID/APOBEC family of genes was evaluated in CD138 + plasma cells from newly diagnosed multiple myeloma (NDMM) patients (*n* = 409), using RNA sequencing; **B** Deaminase activity evaluated in the lysates of CD138 + plasma cells from normal donors (NPC) (*n* = 5), newly diagnosed multiple myeloma patients (*n* = 5), and myeloma cell lines (*n* = 12); **C** Expression of APOBEC3G in CD138 + plasma cells from normal donors (NPC) (*n* = 5), newly diagnosed multiple myeloma patients (*n* = 5), and myeloma cell lines (*n* = 12), evaluated by qRT-PCR. Asterisk indicates a *p* value < 0.05.

We evaluated the expression of APOBEC family of proteins in myeloma patient samples and cell lines. RNA sequencing of CD138 + plasma cells from patients with newly diagnosed MM (*n* = 409) showed that APOBECs 3A, 3B, 3C, and 3G are highly expressed with 3C and 3G being the most expressed in MM (Fig. 1A). Consistently, the evaluation of expression data in MM cell lines also showed that APOBECs 3B, 3C, and 3G as three most expressed APOBEC genes in MM (Supplementary Fig. 1). The APOBEC family of proteins deaminate cytosines to uracil in single-stranded DNA substrates. Using a modified fluorescent oligonucleotide-based assay [21], we measured the deaminase activity in cell lysates from normal plasma cells from healthy donors (NPC), plasma cells from MM patients (MMPC), and myeloma cell lines (MMCL). The deaminase activity was significantly elevated in patient MM cells and MM cell lines, compared to NPC, suggesting significantly elevated APOBEC activity is in myeloma (Fig. 1B). Since APOBEC3G was identified as one of the most expressed APOBECs, we confirmed its expression in MM patient samples and cell lines using quantitative real-time PCR (Fig. 1C).

### Knockdown of APOBEC3G reduces DNA abasic sites and breaks in myeloma cells

**Fig. 2:**
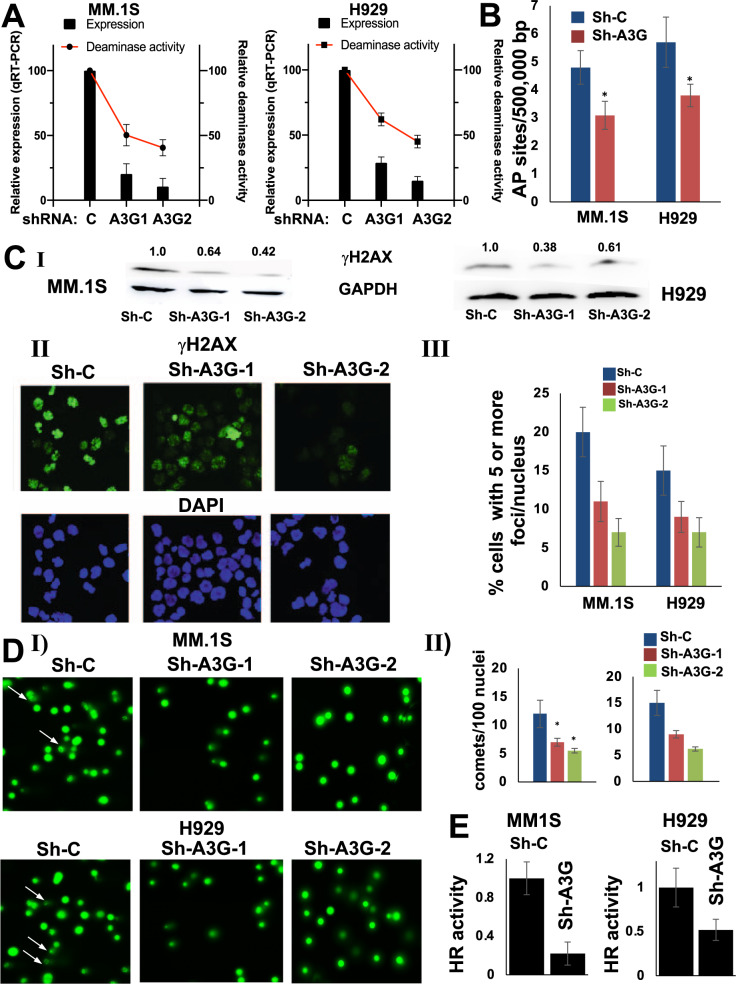
APOBEC3G knockdown reduces DNA breaks and HR activity in myeloma cells. MM.1S and H929 cells were transduced with lentivirus particles carrying control shRNA (Sh-C) or two different shRNAs against APOBEC3G (Sh-A3G-1 and 2) and selected in puromycin. **A** I. Cells were evaluated for A3G expression (by qRT-PCR) and deaminase activity (as described in Methods section); error bars represent SDs of triplicate assays. II. Cells were evaluated for A3G expression by Western blotting. **B** Genomic DNA was isolated from control and A3G knockdown cells and abasic sites were quantified using OxiSelect™ Oxidative DNA Damage Quantitation Kit (AP sites) from Cell Biolabs. **C** I. Western blot analysis of γH2AX in control and A3G knockdown cells; relative expression values of γH2AX (normalized to GAPDH) are shown above corresponding bands. II. Immunofluorescent staining of γH2AX foci in control and A3G knockdown cells. The number of cells with >5 foci/nucleus were quantified (*n* > 100 nuclei) and plotted in the graph. DAPI staining was used to detect nuclei (**D**) DNA breaks in control and A3G knockdown cells evaluated by Comet assay using Cell Biolabs OxiSelect^TM^ Comet Assay kit. The number of nuclei with comets were quantified (*n* > 100 nuclei) and plotted in the graph. **E** Homologous recombination (HR) activity was evaluated in the lysates from control and A3G knockdown MM.1S and H929 cells as described in Methods; and error bars represent SDs of multiple experiments.

To investigate the impact of elevated APOBEC3G expression on genomic integrity, we investigated the cellular impact of shRNA-mediated knockdown of A3G in MM.1S and H929 myeloma cell lines (Fig. 2A). Knockdown of A3G with shRNA1 and shRNA2 resulted in reduction of deaminase activity by 50 ± 6% and 59 ± 8% (*p* < 0.001) in MM.1 S cells and by 38±5% and 65±5% (*p* < 0.007) in H929 cells respectively. We also observed a strong correlation between A3G expression and deaminase activity in both cell lines (*R*^2^ > 0.8), suggesting that A3G significantly contributes to the overall deaminase activity in these cells (Supplementary Fig. 2). Since APOBEC-mediated deamination of cytosine to uracil can lead to generation of abasic sites, we investigated the impact of A3G knockdown in MM cells on generation of abasic sites. A3G-knockdown resulted in 36 and 33% reduction in the number of genomic abasic sites in MM.1S and H929 cells, respectively (*p* < 0.05; Fig. 2B). Since repair of abasic sites involves cleavage of DNA by base excision repair endonuclease, we investigated the impact of elevated A3G on DNA breaks in MM cells by evaluating the expression of γH2AX (a DNA break marker) by both immunofluorescence staining and Western blotting. Knockdown of A3G resulted in reduced γH2AX expression (Fig. 2C, panel I). Consistently, the knockdown of A3G with two different shRNAs reduced the number of γH2AX foci in MM.1S and H929 cells by 45−65 and 40−53%, respectively (*p* < 0.05; Fig. 2C, panel II). In order to further confirm these results, we also performed a single-cell gel electrophoresis (comet assay) to detect DNA breaks. A3G knockdown significantly reduced the DNA damage in MM cells as determined by comet assay [22]. Relative to control cells, A3G knockdown in MM.1S and H929 cells with two different shRNAs reduced the comets by 54−76 and 79−83%, respectively (*p* ≤ 0.05) (Fig. 2D). These data demonstrate that elevated A3G contributes to increased DNA breaks in MM cells.

We have previously demonstrated that elevated/dysregulated homologous recombination (HR) activity is involved in ongoing genomic rearrangements and instability in MM [1]. Since HR can be induced by DNA breaks, we investigated the impact of A3G knockdown on HR activity and observed reduced HR activity by 78 and 48% in A3G knockdown MM1S and H929 cells, respectively (*p* = <0.05) (Fig. 2E). Taken together, these results show that elevated A3G increases DNA damage/breaks and contributes to dysregulation of HR activity in MM cells.

### Knockdown of APOBEC3G reduces genomic instability in myeloma cells

We also evaluated the impact of A3G knockdown on genome stability using various approaches including evaluation of micronuclei (a marker of genomic instability), mutational changes in a plasmid substrate and copy number and genomic changes over time, using whole-genome sequencing.

#### Impact on micronuclei

**Fig. 3:**
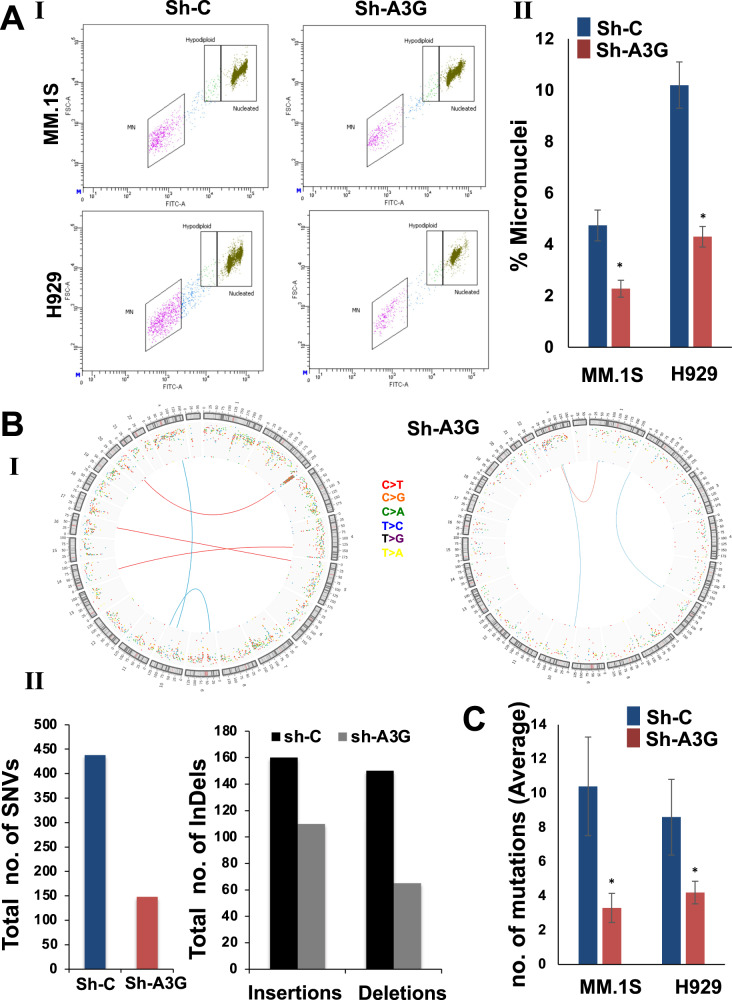
APOBEC3G knockdown reduces genomic instability in myeloma cells. **A** Control (sh-C) and APOBEC3G knockdown (sh-A3G) MM.1S and H929 cells were evaluated for the presence of micronuclei, using In Vitro MicroFlow Kit (Litron Labs). Images of micronuclei (I) and bar graphs (II) showing percentage of micronuclei are presented. **B** MM.1S cells were transduced with control shRNA (sh-C) or that targeting A3G (sh-A3G) and following puromycin selection, cultured for 3 weeks. DNA from these and day 0 (baseline control) cells was extracted and the acquisition of new genomic changes in cultured relative to day 0 cells monitored, using whole genome sequencing (30x). Circos plots showing translocations and other genomic changes (I) and bar graphs showing total number of SNVs and indels (II) are presented. **C** Control and A3G knockdown MM cells were transfected with a plasmid expressing EGFP. EGFP-positive cells were purified, cultured for additional 72 h, genomic DNA isolated, and EGFP sequences amplified using specific primers. The PCR products were cloned into a plasmid vector to transfect into competent *E. coli* cells and plated on LB agar. Plasmid DNA from 10 colonies/sample were isolated and sequenced to analyze for mutations. Bar graphs show APOBEC-like mutations.

Ongoing genomic rearrangements and instability is associated with generation of DNA/chromosomal fragments and formation of micronuclei, which are used as marker of genomic instability [23, 24]. Knockdown of A3G protein resulted in 52 ± 14% and 58 ± 9% reduction in the number of micronuclei in MM.1S and H929 cells, respectively (*p* = <0.05) (Fig. 3A).

#### Evaluation of impact on genomic instability using whole genome sequencing

In order to further evaluate the impact of A3G-knockdown on genome stability, control and A3G-knockdown MM cells were cultured for three weeks and new genomic changes acquired in culture, relative to “day 0” cells (representing baseline genome) were identified, using whole genome sequencing. As seen in Fig. 3B (panel I), the knockdown of A3G resulted in genome-wide reduction in the acquisition of new translocations, SNVs as well as Indels. Throughout genome, the acquisition of SNVs, small insertions and deletions in A3G knockdown cells were reduced by 65%, 30 and 50%, respectively (Fig. 3B, panel II).

DNA samples evaluated by whole genome sequencing (in Fig. 3B), were also investigated using SNP 6.0 array (Affymetrix). The genome of “Day 0” cells was used as baseline to identify new copy-number events in control and A3G-knockdown cells at 3 weeks. As shown in Supplementary Fig. 3A, the copy number events acquired throughout chromosomes were reduced in A3G-knockdown, relative to control cells. Assessment of total copy number change events detected throughout genome (Supplementary Fig. 3B) and on individual chromosomes (Supplementary Fig. 3C) indicate that the acquisition of new genomic changes in A3G knockdown cells, relative to control cells, was reduced by 60%. These data demonstrate that A3G knockdown reduces the acquisition of new genomic changes in MM cells.

#### Evaluation of impact on acquisition of mutations in a plasmid substrate

APOBEC proteins induce deamination of Cytosines in ssDNA converting them to Uracil which usually results in C>T or C>G mutations or much less frequently in C>A mutations. We transduced both control and A3G knockdown cells with a EGFP expressing plasmid. EGFP-positive cells were purified and cultured for additional 72 hr. Genomic DNA was isolated and EGFP sequences amplified using specific primers. The PCR products were purified and cloned into a plasmid vector to transfect into competent *E. coli* cells and plated on LB agar. Plasmid DNA from 10 colonies/sample were isolated and sequenced to analyze for mutations. As shown in Fig. 3C, knockdown of A3G in MM.1S and H929 reduced APOBEC-like mutations (C>T, C>G and C>A) in a plasmid substrate by 68 and 51%, respectively (*p* ≤ 0.05).

### Overexpression of APOBEC3G induces DNA double stand breaks, HR activity, and genomic instability in myeloma cells

**Fig. 4:**
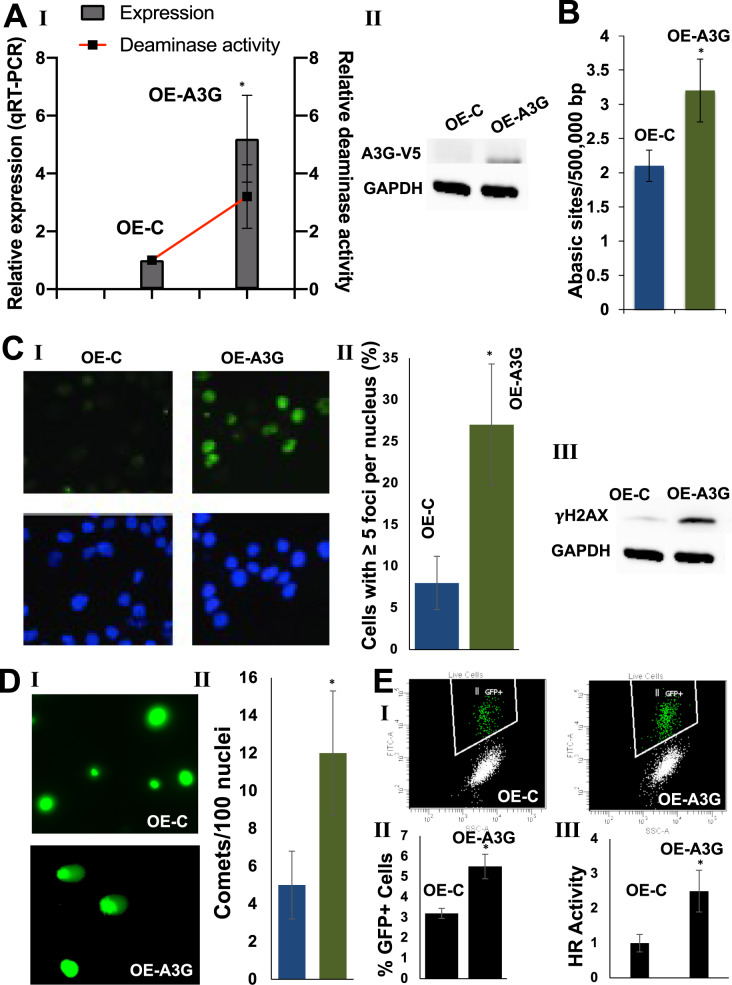
Overexpression of APOBEC3G induces DNA breaks and HR activity in myeloma cells. U266 cells were transduced with lentivirus particles carrying control plasmid (OE-C) or APOBEC3G-ORF with V5 tag (OE-A3G) and selected in puromycin. **A** I. Cells were evaluated for A3G expression by qRT-PCR and in vitro deaminase activity as described in methods; II. Western blot showing A3G expression, evaluated using anti-V5 tag antibody; **B** Genomic DNA from these cells was isolated and abasic sites quantified, using OxiSelect™ Oxidative DNA Damage Quantitation Kit (Cell Biolabs). **C** I–II. Images showing immunofluorescence staining of γH2AX foci and DAPI staining to detect nuclei (I), and the bar graphs showing the number of cells with >5 foci/nucleus (II) in control and A3G overexpression cells; III. Western blot analysis of γH2AX in control and A3G overexpression cells; **D** DNA breaks in control and A3G overexpression cells evaluated by Comet assay, using Cell Biolabs OxiSelect^TM^ Comet Assay kit. Images of Comets (I) and bar graphs showing the number of nuclei with comets in >100 nuclei examined (II) are presented; **E** Homologous recombination (HR) activity was evaluated in control and A3G-overexpressing U2OS cells, using DRGFP assay (I–II), and in the lysates of control and A3G-overexpressing U266 cells using an in vitro assay described in Methods section.

To further confirm the role of APOBEC3G in genomic instability, we used ectopically overexpressed V5 tagged A3G in U266 MM cells that have relatively low endogenous A3G expression. As predicted, these A3G-overexpressing cells has significantly upregulated deaminase activity (Fig. 4A, panel I). The overexpression lead to significant increase in the number of abasic sites by 49% (*p* < 0.05) in A3G-overexpressing cells relative to control cells (Fig. 4B). To monitor the impact of A3G overexpression on DNA breaks, the cells were evaluated for DNA breaks by γH2AX foci using immunofluorescence and γH2AX expression by Western blotting. The percentage of cells with ≥5 γH2AX foci increased by 3.4-fold (*p* ≤ 0.01) in A3G-overexpressing, relative to control cells (Fig. 4C, panels I–II). Commensurate with foci, the γH2AX expression was also increased in A3G-overexpressing cells (Fig. 4C, panels III). Impact of A3G overexpression on DNA breaks was also evaluated using a single-cell gel electrophoresis assay. DNA breaks as measured by comets increased by 2.4-fold; (*p* < 0.05) in A3G-overexpressing cells (Fig. 4D). Thus, evaluation of γH2AX foci, γH2AX expression as well as evaluation by Comet assay indicates that A3G-overexpression increases DNA breaks in MM cells.

#### Impact of A3G overexpression on HR activity and genomic instability

To investigate the impact of A3G-overexpression on HR activity, we used both the MM and U2OS (osteosarcoma) cell lines. U2OS osteosarcoma cells, stably integrated with HR reporter substrate (DRGFP), were transduced with control plasmid or expressing A3G. Transduced cells were then infected with adenovirus expressing I-SceI endonuclease to initiate HR. In this assay system, the HR generates a functional GFP gene resulting in the fluorescence which is detected by flow cytometry. As seen in Fig. 4E (panels I–II), A3G overexpression resulted in 1.7-fold increase (*p* ≤ 0.05) in HR activity in U2OS cells. Consistently, the evaluation of HR activity in MM (U266) cell lysates showed a 2.5-fold increase (*p* ≤ 0.05) in A3G-overexpressing, relative to control cells (Fig. 4E, panel III).

**Fig. 5:**
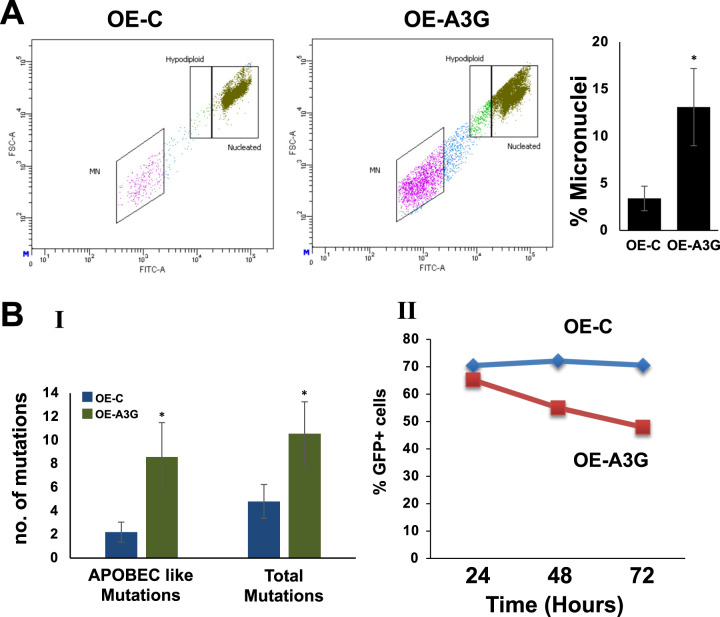
APOBEC3G overexpression induces genomic instability in myeloma cells. Control and A3G-overexpressing U266 cells (described in Fig. 4) were evaluated for genomic instability. **A** Following selection, the cells were evaluated for the presence of micronuclei, using In Vitro MicroFlow Kit (Litron Labs). Images of micronuclei (I) and bar graphs showing percentage of micronuclei (II) are presented. **B** Control and A3G-overexpressing cells described above were transfected with a plasmid expressing EGFP. EGFP-positive cells were purified, cultured for additional 72 h, genomic DNA isolated, and EGFP sequences amplified using specific primers. The PCR products were cloned into a plasmid vector to transfect into competent *E. coli* cells and plated on LB agar. Plasmid DNA from 10 colonies/sample were isolated and sequenced to analyze for mutations. I. Bar graphs show APOBEC-like mutations as well as total (or overall) mutations; II. Percent GFP + cells were analyzed by flow cytometry at different time points and plotted in the graph.

We also observed significant increase (3.8-fold; *p* ≤ 0.05) in micronuclei in A3G-overexpressing U266 cells relative to control cells (Fig. 5A). To further investigate the effect of A3G overexpression on the acquisition of mutations, we stably transduced a EGFP expressing plasmid in control and A3G-overexpressing U266 cells. EGFP positive cells were purified using cell sorter and cultured for additional 72 h. Genomic DNA was isolated, and EGFP sequences were amplified and sequenced as in Fig. 3C. Overexpression of A3G increased the number of APOBEC-like (C>T, C>G, and C>A) mutations by 3.9-fold (*p* ≤ 0.05) in the EGFP plasmid substrate (Fig. 5B). We also observed a 2.2-fold increase in overall mutations in the EGFP plasmid in A3G-overexpressing cells compared to control cells. However, the number of overall mutations is not significantly different than the APOBEC-like mutations, suggesting that A3G-overexpression predominantly caused APOBEC-like (C>T, C>G, and C>A) mutations in the plasmid substrate. These data demonstrate that A3G overexpression is associated with increased mutational instability. We also investigated if this increased mutation rate could lead to the loss of EGFP signal. In fact, as seen in (Fig. 5B, panel II), the A3G overexpression resulted in the loss of EGFP + cells over time, suggesting that overexpression of APOBECs can cause deleterious mutations leading to loss of function.

Overall, using various approaches, we demonstrate that A3G knockdown significantly inhibits whereas its overexpression increases abasic sites, DNA breaks, HR activity, and genomic instability in MM cells.

## Discussion

Genome instability is one of the prominent features observed early at MGUS stage and persists and may even be enhanced on progression to MM [25–27]. Genomic instability underlies the development of clonal heterogeneity in MM which is associated with disease progression including development of drug resistance and relapse. The factors contributing to genomic instability in cancer may include increased DNA damage [28], dysregulated DNA repair [1, 29], replication stress [30], oxidative stress [31], and mitotic segregation errors [32]. The molecular mechanisms and sequence of events driving genomic instability and clonal evolution in cancer are not fully understood, and understanding these mechanisms has important implications in devising translational strategies to treat and/or prevent cancer. Previously we have demonstrated that homologous recombination (HR) is dysregulated in MM and contributes to genomic instability and drug resistance [1]. Investigating the mechanisms underlying dysregulated HR, we reported base excision repair related apurinic/apyrimidinic (APEX) nucleases to contribute to regulation of recombinase RAD51 and HR in MM [29]. Moreover, the whole genome sequencing data has also identified APOBEC contributing mutations associated with progression to MM with further increase at relapse. Since APOBEC-mediated deamination of cytosine to uracil can lead to generation of abasic site, the substrate of APEX activity, we investigated the role of specific APOBEC in genomic instability.

Here we report that several members of APOBEC3 family (A3A, A3B, A3C, and A3G) are highly expressed in MM. Consistently, the APOBEC deaminase activity was also elevated in MM cells and cell lines, relative to normal plasma cells. In specific, A3G, is one of the most expressed APOBEC genes in MM. In normal cells, A3G is predominantly found in the cytoplasm where it acts as an innate immune barrier against viral infections, targeting viral DNA [33]. APOBEC3G has been extensively studied in the context of its anti-viral functions inhibiting the infectivity of HIV, hepatitis B, human T cell leukemia virus type 1, and human papillomavirus [34–38]. APOBEC enzymes exert their anti-viral activity by targeted deamination of cytidine residues in the ssDNA produced during reverse transcription of viral genomic RNA. However, it has been shown that DNA damage could trigger the translocation of A3G to the nucleus and A3G has been shown to inhibit retro-transposition of endogenous retroviruses. A3G is constitutively expressed in immune cells and is further induced by interferon (IFN) [39]. With regards to cancer, A3G has been shown to promote liver metastasis in an orthotopic mouse model of colorectal cancer [40], sensitize mesenchymal gliomas to radiation-induced cell death [41], and enhance lymphoma cell radio resistance by promoting cytidine deaminase-dependent DNA repair [42, 43].

Importantly, the role of APOBEC has been imputed in the development of number of malignancies, especially MM. Early studies evaluating progression of SMM to MM, identified APOBEC activity as the key late event shaping the progressor phenotype to MM [4]. Similarly, early Exome sequencing identified APOBEC driven mutational signature as one of the 2 key events in MM [3]. A study evaluating mutational profile in newly diagnosed MM using deep whole genome sequencing identified APOBEC mutational events shaping the landscape of later stages of MM [44]. This study utilized clonality of the mutations and signatures driving those mutations to recreate the evolution of the disease and described that especially in the high-risk group, the APOBEC signature played a predominant role in the middle phase of progression. This study also identified differential utilization of various mutational processes in various subgroups and reported that the APOBEC-related mutational process was significantly higher in t(14;16) MM. Interestingly, t(14;16) MM has the highest mutational burden which is associated with poor survival outcome. This adverse role of APOBEC-mediated mutational signature was also confirmed by an independent large study reporting association with poor prognosis primary and secondary translocations [6].

Suppression of A3G in MM cells significantly reduced, whereas its overexpression increased the number of abasic sites. Since APOBEC-mediated deamination of cytosine to uracil can lead to generation of abasic site, this result is consistent with the functional nature of APOBEC proteins. Abasic sites are repaired by base excision repair related APEX nucleases, which cleave the DNA 5′ to the abasic site [45]. Our previous studies have demonstrated that APEX nucleases are overexpressed and contribute to increased DNA breaks (unpublished data) and HR activity in MM cells [29]. Results here with A3G are consistent and in line with the previous observation and indirectly provides a relationship to a proximal pathway impacting genomic integrity in MM. The observation that suppression of A3G significantly reduced, whereas its overexpression increased DNA breaks in MM cells suggest that A3G-induced abasic sites could at least partly be attributed to the observed upregulation of APEX nucleases in MM cells [29]. The results here also show a link between elevated A3G and increased HR activity using two different HR assay systems in 2 different cell types. One possible interpretation of these results is that increased DNA breaks by elevated A3G expression increase the need for HR-mediated repair. DNA breaks are known to induce HR, which has been shown to be dysregulated and involved in the acquisition of ongoing genomic rearrangements in MM [1]. Alternatively, A3G could also play a direct role in homologous recombination. Evaluation in lymphoma cells has shown that A3G promotes repair of ionizing radiation-induced double strand breaks mainly through non-homologous end joining [42]. However, a role of A3G in HR has not been reported previously. To our knowledge, this study is the first report establishing a possible link between A3G and HR in myeloma cells. Although a direct role of A3G in HR cannot be ruled out, it needs to be investigated further.

Due to the low sample size, we were not able to detect specific mutational signatures in this investigation. However, we do demonstrate the impact of A3G modulation in a plasmid substrate showing that the knockdown of A3G reduced, whereas its overexpression increased, the incidence of overall as well as APOBEC-like (C>T, C>G, and C>A) mutations (Figs. 3B and 5B). These data demonstrate that in addition to known C>T, C>G, and C>A mutations, A3G could probably also contribute to other mutation types in myeloma cells. Taken together, these data demonstrate that elevated A3G contributes to dysregulation of HR and genome stability.

**Fig. 6:**
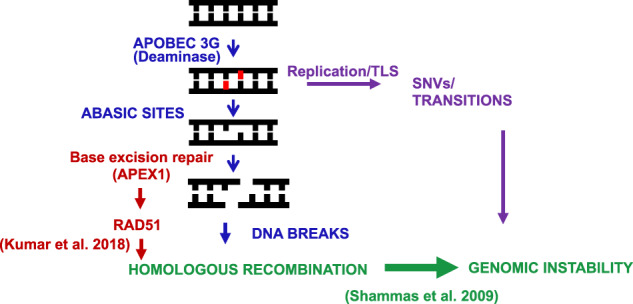
Proposed model for the role of APOBEC3G in genomic instability in multiple myeloma. Elevated A3G-induced deaminase activity leads to generation of abasic sites. Processing of increased abasic sites then leads to increase in DNA breaks. Previous data from our laboratory demonstrate that APEX1, the nuclease which is involved in the repair of abasic sites by cutting the DNA 5′ to abasic site, is elevated and contributes to dysregulation of RAD51 and homologous recombination [29], a mechanism of genomic instability in MM [1]. Increased abasic sites by A3G and the replication stress caused by abasic sites can also contribute to genomic instability by increasing the likelihood of translesion synthesis [50].

These data, in light of our previous observations, suggest a significant role of A3G in genomic instability in MM cells (Fig. 6). We propose that the elevated A3G-induces deaminase activity leading to increased generation of abasic sites with subsequent increase in DNA breaks and HR activity. Moreover, a direct role of A3G in dysregulation of HR in multiple myeloma and its additional roles in genomic instability cannot be ruled out and requires further investigation. These data also identify A3G as a novel target to inhibit/reduce DNA damage, HR activity, and genomic instability in MM cells.

## Materials and methods

### Cell lines and patient samples

Multiple Myeloma (MM) cell lines were purchased from the American Type Tissue Culture Collection (Rockville MD). Cell lines were cultured in RPMI1640 medium containing 10% fetal bovine serum and antibiotic and maintained in logarithmic growth. Samples of bone marrow aspirates from myeloma patients and normal individuals were obtained following informed consent under the protocol approved by Institutional Review Board of Dana Farber Cancer Institute. MM cells were isolated by Magnet Assisted Cell Sorting (MACS, Miltenyi Biotech), according to the manufacturer’s protocol.

### Western blotting, immunocytochemical detection of proteins, and antibodies used

For Western blotting, lysates boiled in sample buffer, were fractionated on gradient SDS-polyacrylamide gel and subsequently electroblotted onto nitrocellulose paper. The blots were incubated with indicated primary antibodies, washed and incubated in either anti-rabbit or anti-mouse, horseradish peroxidase conjugates. After washing, specific proteins were detected using an enhanced chemiluminescence, according to the instructions provided in the manual (Amersham Life Sciences Inc., Arlington Heights, IL). For immunocytochemical detection of γ-H2AX, cytospins of normal plasma cells and MM cells were fixed in methanol/acetone (1:1) for 10 min at –20 °C. Fixed cells were rinsed, rehydrated in PBS, and incubated with mouse monoclonal antibody to γ-H2AX (Ser139), clone JBW301 (Catalog #05–636; Millipore). Other antibodies used were anti-GAPDH (14C10) HRP conjugated antibody (Catalog #3683; Cell Signaling Technology), anti-V5 tag antibody SV5-Pk1 (Catalog #ab27671; Abcam), anti-rabbit IgG, HRP-linked antibody (Catalog #7074; Cell Signaling Technology) and anti-mouse IgG, and HRP-linked antibody (Catalog #7076; Cell Signaling Technology).

### Detection of DNA breaks

DNA breaks were estimated by evaluating the levels of γ-H2AX (DNA break-associated protein) and Comet, a gel-based assay for the detection of DNA breaks. Levels of γ-H2AX protein were measured by immunofluorescence staining and/or by quantification of γ-H2AX by Western blotting. Comet assay was performed using OxiSelect™ Comet Assay Kit (Cell Biolabs Inc., San Diego, CA) following manufacture’s protocol.

### DNA cytidine deaminase activity assay

Cytidine deaminase activity was measured in the myeloma cell lysates using a fluorescent oligonucleotide-based assay as described before [21]. Briefly, cell lysates were incubated at 37 °C for 2 h in the presence of a single-stranded (ss) DNA oligonucleotide 5′(6-FAM) AAATTCTAATAGATAATGTGA (TAMRA)3′. In the absence of deaminase activity, the fluorescence is quenched by TAMRA. In the presence of cytidine deaminase activity in the lysates, cytidine is converted to uridine. This is subsequently excised by the addition of Uracil DNA glycosylase (UDG) resulting in the creation of abasic sites. The abasic sites are cleaved by incubation at 95 °C resulting in the increased fluorescence signal, which is measured using a fluorescence plate reader.

### HR activity assays

Homologous recombination (HR) activity was assessed either in cell lysates, using in vitro HR conditions [46] as described previously [29] or in intact cells, using a fluorescence-based HR substrate (Addgene, Cambridge, MA) [47] as described by us previously [48].

### Micronucleus assay

To evaluate the impact on genomic instability, control and A3G knockdown cells were analyzed for micronuclei, using a flow cytometry-based Micronucleus Assay (MicroFlow kit, Litron Laboratories, New York, USA) as described by us previously [29].

### Evaluation of impact on mutations in a plasmid substrate

Control and A3G-knockdown or A3G-overexpression cells were transduced with lentivirus particles carrying EGFP expression vector. GFP positive cells were purified, using flow cell sorter and cultured for additional 72 h. Genomic DNA was isolated using PureLink Genomic DNA Mini Kit from Thermofisher. EGFP sequences were PCR amplified, using specific primers. Purified PCR products were cloned into plasmid vector, transfected into competent *E. coli* cells and grown on LB agar plates overnight. Plasmid DNA was isolated from 10 individual colonies per sample and sent for sequencing. The number of APOBEC-like (C>T, C>G, C>A) conversions were quantified and the average number of mutations were plotted in the graph.

### Evaluation of impact on genomic instability using SNP/WGS

Control and transgenically-modulated cells were cultured for three weeks. Live cells were separated, using Ficoll density gradient centrifugation and genomic DNA from these and “day 0 cells” (representing baseline genome) was isolated, using PureLink Genomic DNA Mini Kit, and analyzed using single nucleotide polymorphism (SNP6.0) arrays (Affymetrix) or whole-genome sequencing (WGS). For both the SNP and WGS experiments, the genome of “day 0” cells (harvested and stored in the beginning of each experiment) was used as baseline to identify the changes in control and transgenically-modulated MM cells that were cultured for three weeks. SNP and WGS data were analyzed as reported previously [1, 4].

### Statistics and reproducibility

RNA Sequencing data were analyzed as described previously [49]. Experiments were conducted in triplicate and bar graphs with error bars indicating SD values presented; two-tailed *p* values were derived by Student’s *t* test. To confirm the genomic impact of APOBEC3G, both the gain and loss of function, a total of three MM cell lines and the evaluation of different parameters of genome maintenance, using multiple approaches, were used. The overexpression of APOBEC3G in one MM cell line and its knockdown in two MM cell lines were evaluated for impact on deaminase activity, abasic sites, spontaneous DNA breaks (using Western blotting, immunofluorescence and Comet assay), homologous recombination activity and genomic instability (using micronucleus assay, and evaluation of the acquisition of genomic changes over time using single nucleotide polymorphism arrays and whole-genome sequencing).

## Supplementary information

Supplementary Material
