# Supplementary material for: Dysregulated APOBEC3G causes DNA damage and promotes genomic instability in multiple myeloma
Source: Blood Cancer J. 2021 Oct 8;11(10):166. doi: 10.1038/s41408-021-00554-9 (PMC8501035; doi:10.1038/s41408-021-00554-9)
Supplement: Supplementary file 1 — Supplementary Material [file 41408_2021_554_MOESM1_ESM.pdf]

## Supplementary Figure 1

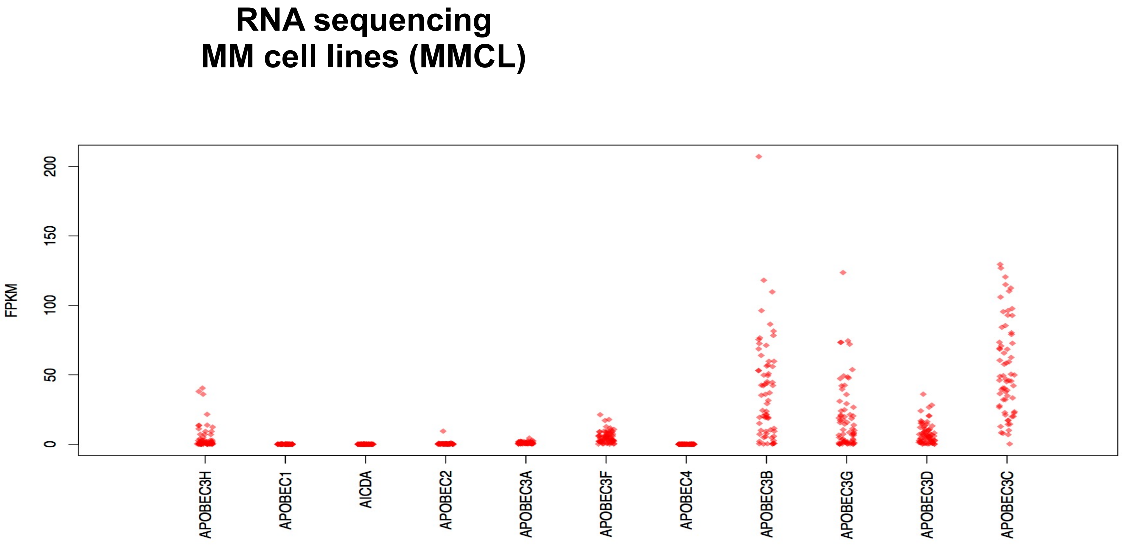


**Supplementary Figure 1. Relative expression of AID/APOBEC family of genes in myeloma cell lines.** Log2(TPM+1) expression of different AID/APOBEC family members in myeloma cell lines, evaluated by RNA sequencing.

Supplementary Figure 2

**I** **MM.1S**

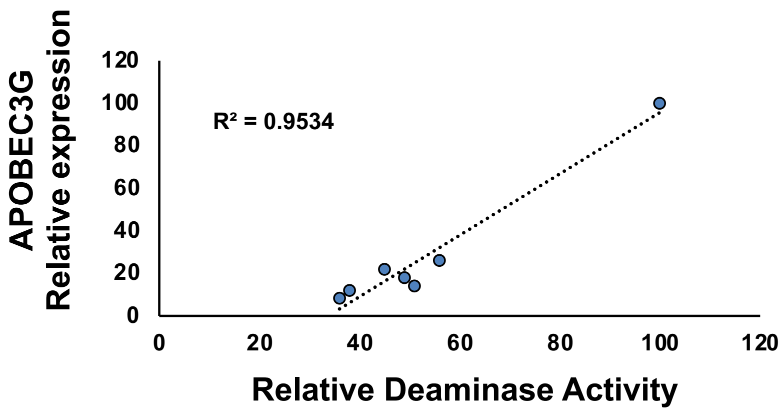


**II** **H929**

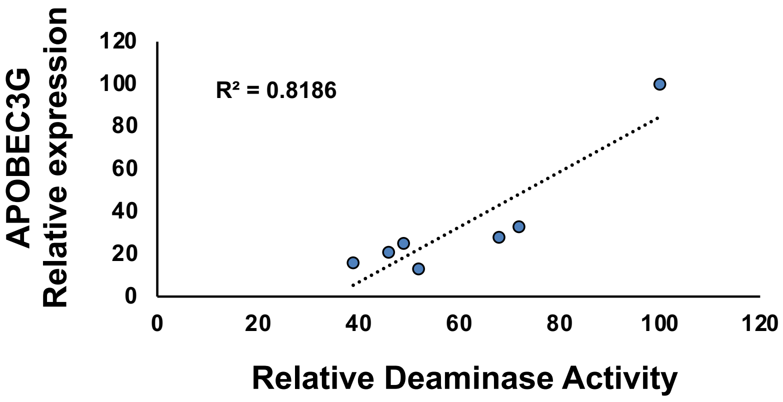


**Supplementary Figure 2. Correlation between expression of APOBEC3G and deaminase activity.** MM.1S (I) and H929 (II) cells were treated with control shRNA or two different shRNAs targeting A3G, in triplicate. For each of these six treatments and the average of control shRNAs, the A3G expression and deaminase activity were evaluated and plotted. Line plots show that A3G expression correlates with deaminase activity.

Supplementary Figure 3

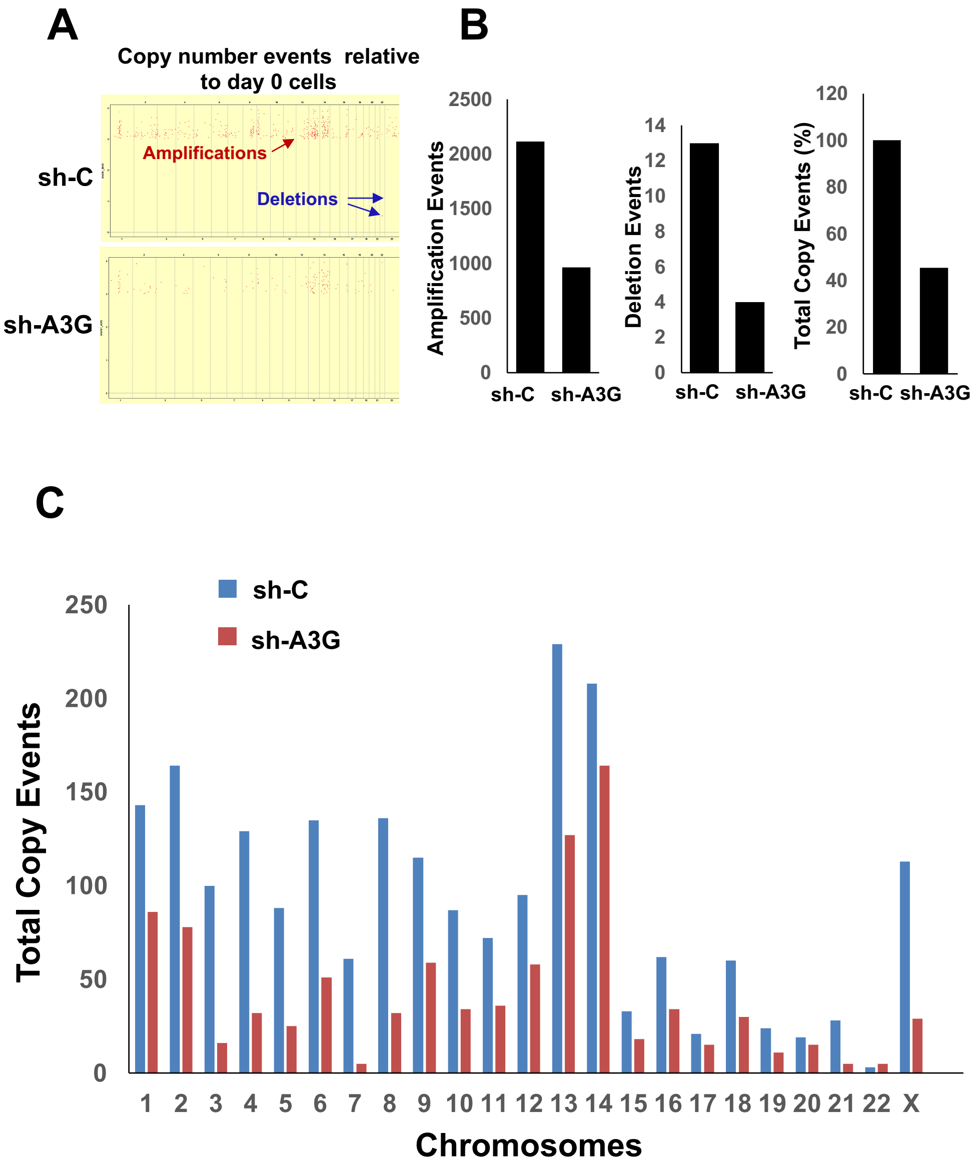


**Supplementary Figure 3. A3G knockdown in MM cells reduces the acquisition of new copy number changes over time.** Myeloma (MM.1S) were transduced with control shRNA (sh-C) or that targeting A3G (sh-A3G) and following puromycin selection, cultured for three weeks. DNA from these and day 0 (baseline control) cells was extracted and the acquisition of copy number events in cultured relative to day 0 cells monitored, using SNP6.0 arrays (Affymetrix). A copy number event was defined as a change in ≥ 3 consecutive CNV probes by 1 copy. (A) Images show the copy number events acquired by sh-C and sh-A3G cells in three weeks, relative to day 0 cells. throughout chromosomes. Amplifications (red dots) and deletions (blue dots) are shown; (B-C) Bar graphs showing copy-number change events, throughout genome (B) or on individual chromosomes (C).
